# Peptide-functionalized NaGdF_4_ nanoparticles for tumor-targeted magnetic resonance imaging and effective therapy†

**DOI:** 10.1039/c9ra02135c

**Published:** 2019-05-31

**Authors:** Yixin Chen, Yu Fu, Xiaodong Li, Hongda Chen, Zhenxin Wang, Huimao Zhang

**Affiliations:** Department of Radiology, The First Hospital of Jilin University Changchun 130021 P. R. China huimaozhanglinda@163.com +86-431-88783300; State Key Laboratory of Electroanalytical Chemistry, Changchun Institute of Applied Chemistry, Chinese Academy of Sciences Changchun 130022 P. R. China chenhongda@ciac.ac.cn +86-431-85262756

## Abstract

Metallic nanoparticles showed potent efficacy for diagnosis and therapy of cancer, but their clinical applications are limited by their poor tumor-targeting ability. Herein, peptide-functionalized 9 nm NaGdF_4_ nanoparticles (termed as, NaGdF_4_@bp-peptide NPs) have been synthesized through the Gd–phosphate coordination reaction of the spherical NaGdF_4_ nanoparticles with phosphopeptides (sequence: KLAKLAKKLAKLAKG(p-S)GAKRGARSTA, p-S means phosphorylated serine) including a p32 protein binding motif incorporating a cell-penetrating function, and a proapoptotic domain. The NaGdF_4_@bp-peptide NPs are ready to be efficiently internalized by cancer cells; they show a much higher cytotoxicity in MCF-7 breast cancer cells than the casein phosphopeptide (CPP) modified NaGdF_4_ nanoparticles (termed as, NaGdF_4_@CPP NPs). Using mouse-bearing MCF-7 breast cancer as a model system, the *in vivo* experimental results demonstrate that NaGdF_4_@bp-peptide NPs have integration of T_1_-weighted magnetic resonance imaging (MRI) contrast and tumor-targeting functionalities, and are able to suppress tumor growth without causing systemic toxicity.

## Abstract 2

NaGdF_4_@bp-peptide nanoparticles have been used as a T_1_-weighted MR contrast agent with active-tumor targeting and antitumor ability.

## Graphical abstract

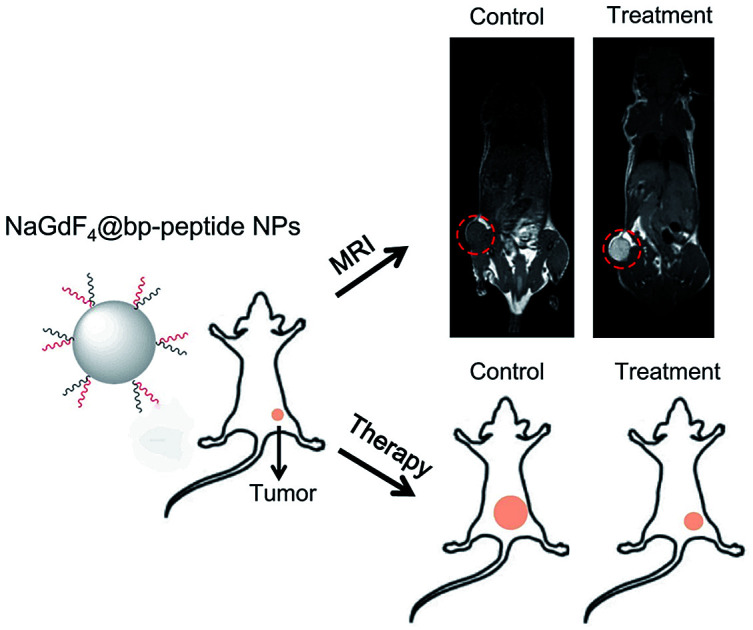

## Introduction

Nanotheranostics (also known as nanomedicines), integrating diagnostic and therapeutic functions into one nanosized material, have many advantages over the conventional molecular medicines, such as prolonged blood circulation time, controlled clearance pathways, and tunable physical properties for multimodal imaging.^1–7^ To date, numerous nanomaterials, including inorganic nanoparticles, organic nanoparticles, nanohydrogels, and organic–inorganic hybrid nanomaterials have been developed as promising nanotheranostics for precise treatment of cancer (as well as many other diseases). Although a few nanotheranostics have been approved by the U. S. Food and Drug Administration (FDA) for drug delivery (*e.g.*, Doxil and Abraxane) or contrast agents (*e.g.*, Feridex IV) for magnetic resonance imaging (MRI), clinical translation efforts of have been hampered by their low tumor delivery efficiency.^8–10^ For instance, Chan and co-authors found that only 0.59% injected dose of trastuzumab-coated nanoparticles reached the mouse-bearing SKOV-3 xenograft tumor through intravenous administration.^10^ The tumor delivery efficiency of nanomaterials is strongly determined by their own physicochemical properties including size, surface modification, and stimuli-responsiveness to tumor microenvironment.^11–16^ Therefore, there is a strong desire for the development of new modification strategies for generating nanomaterials with improved diagnostic and therapeutic capabilities through increasing their tumor accumulating amounts.

Because of its low ionizing radiation, magnetic resonance imaging (MRI) enables noninvasive detecting and assessing disease progression at relatively frequent time intervals.^17–19^ However, it has been clinically found that the sensitivity of MRI is poor, resulting in difficulty in accurate diagnosis of tumor at early stage. In order to overcome detection limitation of MRI, contrast agents (CAs) are usually used for improvement of the signal-to-noise ratios (SNRs) in the imaging process through altering the relaxation time of local water molecules in lesion regions.^20,21^ Gadolinium (Gd) chelates (*i.e.*, Gd-DTPA and Gd-DOTA) are currently clinical used CAs, which suffer low tumor accumulation efficiency and short circulation time. Benefiting from their unique physicochemical properties, the application of nanoparticles in bioimaging field has been demonstrated as a very promising strategy for circumventing the inherent defects of traditional small molecular CAs in terms of bioavailability and targeting ability.^21–30^ Versatile nanoparticle-based CAs have been constructed for MRI, computed tomography (CT), photoacoustic imaging (PAI) and fluorescence imaging (FI) to enhance the spatial resolution and detection sensitivity of these bioimaging modes. For example, nanoparticles containing Gd such as gadolinium oxide (Gd_2_O_3_) nanoparticles and sodium tetrafluoro gadolinium (NaGdF_4_) nanoparticles are appropriate CAs of MRI for visualization of tumor details and/or MRI-guided therapy of tumors.^31,32^

Peptides are one kind of important biological materials, which normally present structure-dependent functions. Peptides and peptide derivatives have been extensively employed for constructing multifunctional nanotheranostics since they exhibit chemical versatility and enable to specifically recognize other biomacromolecules.^33–51^ In particular, conjugation of nanoparticles with tumor-homing peptides and/or therapeutic peptides can generate novel nanotheranostics for highly sensitive tumor imaging and effective tumor-targeted therapy. For example, Ruoslahti and coauthors have been developed a theranostic nanosystem, which consists of iron oxide nanoworms conjugated with a composite peptide with proapoptotic domain (*i.e.*, therapy motif) and cell surface p32 protein binding domain (*i.e.*, tumor-homing motif).^52,53^ The *in vivo* experimental results indicate that the as-prepared theranostic nanosystem has excellent homing and penetrating activity in mouse-bearing breast tumor models, and enables to effectively retard tumor growth. Very recently, we also synthesized a kind of peptide mixture-modified NaGdF_4_ nanodot with active tumor targeting ability, which can be used as high efficient T_1_-weighted MRI CA for tracking small drug induced orthotopic colorectal tumor (*c.a.*, 195 mm^3^ in volume) in mouse.^54^

Herein, a tumor-specific multifunctional nanotheranostic composed of NaGdF_4_ nanoparticle together with phosphopeptides has been developed for T_1_-weighted MRI guided cancer therapy. Strategically, the p32 protein binding peptide (sequence: AKRGARSTA) and the pro-apoptotic peptide (sequence: KLAKLAKKLAKLAK) were linearly linked together to form a new peptide (sequence: KLAKLAKKLAKLAKG(p-S)GAKRGARSTA, named as bp-peptide) through the phosphorylated tripeptide (–G(p-S)G–: –glycine–phosphorylated serine–glycine–). Benefiting from the strong coordination reaction between Gd(iii) ion and phosphate, the hydrophilic bp-peptide conjugated NaGdF_4_ nanoparticles (NaGdF_4_@bp-peptide NPs) can be easily achieved through replacing initial hydrophobic oleate ligand by the bp-peptide molecules due to the phosphopeptides can be selectively enriched by the rare-earth materials through the coordination reactions of rare-earth ions with phosphate moieties in the peptides.^54,55^ Both of *in vivo* and *in vitro* experimental results demonstrated statistically significant improvement in tumor-targeted uptake and tumor suppression of NaGdF_4_@bp-peptide NPs over these of passive tumor-targeting the casein phosphopeptide (CPP) modified NaGdF_4_ nanoparticles (NaGdF_4_@CPP NPs).

## Experimental section

### Materials

Tryptone (casein phosphopeptide, CPP), 1-octadecene (ODE, 90%), oleic acid (OA, 90%) were obtained from Sigma-Aldrich Co. (St. Louis, USA). The Gd_2_O_3_ (99.99%) were reacted with excess HCl (6.0 mol L^−1^) to form GdCl_3_ solution. After dried completely at 30 °C, the resulting powder was redispersed in H_2_O to yield GdCl_3_ stocking solution (1.5 mol L^−1^). DMEM basic medium and fetal bovine serum (FBS) were purchased from Gibco Co. (New York, USA). Trypsin–EDTA digest and 3-(4,5-dimethylthiazol-2-yl)-2,5-diphenyltetrazolium bromide were purchased from Beijing Dingguo Biotechnology Ltd. (Beijing, China). The peptides were synthesized by Shanghai Synpeptide Ltd. (Shanghai, China). Other reagents (analytical grade) were purchased from Beijing Chemical Reagent Co. (Beijing, China). Deionized H_2_O (18.2 MΩ cm) were used in all experiments. All animal procedures were performed in accordance with the Regulations for the Administration of Affairs Concerning Experimental Animals of the People's Republic of China and approved by the Animal Ethics Committee of Jilin University. The mice had free access to food and water and were raised on a 12 h light/12 h dark cycle at 20 °C.

### Synthesis of NaGdF_4_ nanoparticles

NaGdF_4_ nanoparticles were synthesized by literature reported procedure with slight modification.^54^ Briefly, 1 mL GdCl_3_ stocking solution was dried at 100 °C. Subsequently, 6 mL OA and 22.5 mL ODE were added and well mixed at 160 °C in Ar atmosphere. After cooled to 60 °C, 15 mL CH_3_OH solution containing 6 mmol NH_4_F and 3.75 mmol NaOH were added dropwise, and vigorously mixed for 12 h. After evaporating CH_3_OH completely at 90 °C, the temperature of mixture was slowly (10 °C per minute) increased to 250 °C and maintained for 10 minutes in Ar atmosphere. After cooling to room temperature, the as-prepared NaGdF_4_ nanoparticles were purified by repeated centrifugation (10 000 rpm, three times) and redispersed in 10 mL cyclohexane.

### Ligand-exchange of NaGdF_4_ nanoparticles

12 mL NaGdF_4_ nanoparticle cyclohexane solution (1 mg mL^−1^) were vigorously mixed with 32 mL peptide (bp-peptide or CPP) aqueous solution (2 mg mL^−1^) and stirred at room temperature for 12 h. The aqueous solution was separated, and the peptide modified NaGdF_4_ nanoparticles (NaGdF_4_@bp-peptide NPs or NaGdF_4_@CPP NPs) were purified by centrifugation repeated centrifugation (10 000 rpm, three times) and redispersed in H_2_O.

### Cytotoxicity evaluation

Human breast cancer cells (MCF-7) and normal kidney tissue cells (293) were purchased from the Shanghai Cell Bank of the Chinese Academy of Sciences. The MCF-7 cells and 293 cells were co-cultured with various concentrations of NaGdF_4_@bp-peptide NPs and NaGdF_4_@CPP NPs in DMEM (containing 10% (v/v) FBS and 100 U mL^−1^ penicillium–streptomycin) for 24 h, respectively. Then, the cells were washed and the MTT assays were used to detect cell viabilities. The normally cultured cells were used as control groups.

### *In vitro* study

The MCF-7 cells were co-cultured with various concentrations of NaGdF_4_@bp-peptide NPs and NaGdF_4_@CPP NPs in DMEM (containing 10% (v/v) FBS and 100 U mL^−1^ penicillium–streptomycin) for 4 h, respectively. Subsequently, the cells were washed with fresh culture medium and PBS (10 mM phosphate buffer (PB) with 137 mM NaCl, pH 7.4), respectively. The cells were digested by 1 mL trypsin (0.25 wt% in PBS) solution and collected by centrifugation (2000 rpm, 5 min), respectively. The NaGdF_4_@bp-peptide NPs and NaGdF_4_@CPP NPs stained cells were immobilized in 1 wt% agarose for *in vitro* MRI. The MRI was recorded with the Siemens 3.0 T MRI scanner (0.5 mm (slice thickness), 15 ms (TE), 358 ms (TR), 0.8 mm (in-plane resolution), and 50 mm × 50 mm (field of view)). After treated by 2 mL aqua regia, the amounts of Gd element in the NaGdF_4_@bp-peptide NPs and NaGdF_4_@CPP NPs stained cells were measured by inductively coupled plasma mass spectrometry (ICP-MS, ELAN 9000/DRC, PerkinElmer Co., USA), respectively.

### Biocompatibility analysis

Kunming mice with average body weight of 35 g were ordered from Liaoning Changsheng Biotechnology Ltd. (Liaoning, China). The mice were randomly divided into three groups: control group, NaGdF_4_@bp-peptide NPs treated group and NaGdF_4_@CPP NPs treated group. The mice in treated groups were injected intravenously with 100 μL NaGdF_4_@bp-peptide NPs or NaGdF_4_@CPP NPs (10 mg Gd per kg) in PBS through tail vein, respectively, while the mice in control group were only injected intravenously with 100 μL PBS. The bloods were taken for routine blood tests at 24 h post-injection. The body weights of mice were measured every 2 days until 31 days after injection. The mice were sacrificed, and main organs including heart, liver, spleen, lung and kidneys were collected for hematoxylin–eosin (H&E) staining analysis.

### *In vivo* study

Balb/c nude mice with average body weight of 20 g were purchased from Beijing Vital River Laboratory Animal Technology Ltd. (Beijing, China). The tumor model was constructed by subcutaneous injection of MCF-7 cells (1 × 10^6^ cells in 100 μL PBS). The tumor size (V) was evaluated by the following formula: *V* = length × (width)^2^/2; the inhibition rate of tumor growth (IRT) was calculated as follows: (1 − *V*_NPs_/*V*_Ctrl_) × 100% (*V*_NPs_: tumor volume of NaGdF_4_@bp-peptide NPs treated mouse; *V*_Ctrl_: tumor volume of PBS treated mouse). The MCF-7 tumor-bearing Balb/c nude mice were anesthetized with 100 μL chloral hydrate (10 v/v%) while the size of tumor reached about 400 mm^3^ in volume. Subsequently, the mice were treated with 100 μL NaGdF_4_@bp-peptide NPs or NaGdF_4_@CPP NPs (10 mg Gd per kg) in PBS through intravenous injection *via* the tail vein, respectively. MRI of nude mice were performed at pre-injection (0 h), 1 h, 2 h, 4 h, 8 h and 24 h post-injection by Siemens 3.0 T MRI scanner with the following parameters: TR, 358 ms; TE, 15 ms; field of view, 120 mm × 72 mm and slice thickness, 2.0 mm. In addition, three mice were sacrificed at 24 h post-injection, and main organs and tumors were collected for ICP-MS measurement.

### Treatment efficiency evaluation

9 MCF-7 tumor-bearing nude mice were randomly divided into 3 groups which were treated by 100 μL PBS only (control group), 100 μL PBS containing NaGdF_4_@bp-peptide NPs (10 mg Gd per kg, NaGdF_4_@bp-peptide NPs treated group) and 100 μL PBS containing NaGdF_4_@CPP NPs (10 mg Gd per kg, NaGdF_4_@CPP NPs treated group) through tail vein, respectively. The tumor sites of mice were measured every 2 days until 31 days after injection.

## Result and discussion

### Synthesis and characterization of nanoparticles

**Fig. 1:**
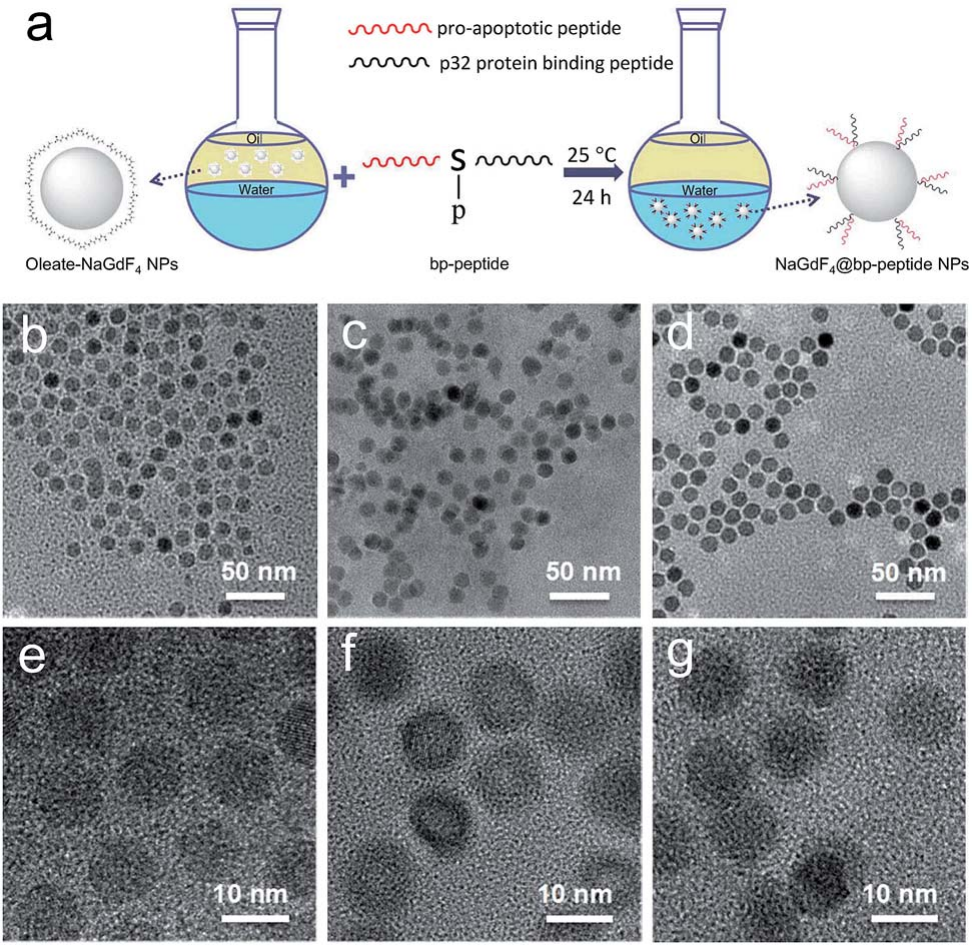
The schematic representation of transfer and functionalization of OA-coated NaGdF_4_ nanoparticles by bp-peptide (a), and TEM and HRTEM of NaGdF_4_ nanoparticles (b and e), NaGdF_4_@bp-peptide NPs (c and f) and NaGdF_4_@bp-peptide NPs (d and g), respectively.

**Fig. 2:**
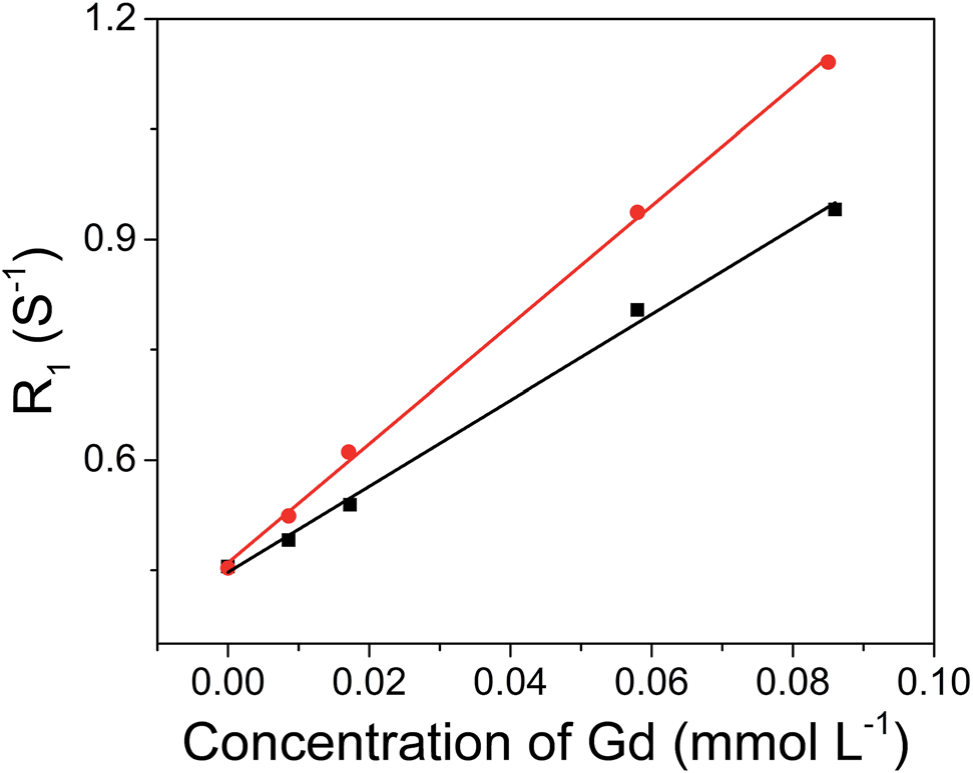
*r*_1_ relaxivities of NaGdF_4_@bp-peptide NPs (black line, *r*_1_ = 5.8 mM^−1^ s^−1^) and NaGdF_4_@CPP NPs (red line, *r*_1_ = 7.9 mM^−1^ s^−1^) as a function of the molar concentration of Gd^3+^ in the solution.

The hydrophobic OA-coated NaGdF_4_ nanoparticles (9.0 ± 1.0 nm in diameter) were synthesized by literature reported strategy with slight modification.^54–56^ In this case, the phosphopeptides (sequence: KLAKLAKKLAKLAKG(p-S)GAKRGARSTA, named as bp-peptide) were employed as a bifunctionalized ligand and phase transfer agent for preparing hydrophilic NaGdF_4_ nanoparticles (NaGdF_4_@bp-peptide NPs), and through formation of Gd^3+^–phosphate coordination bond in the ligand exchange reaction under mild experimental conditions because phosphopeptides have the ability to react with multivalent cations and form robust metal–phosphate coordination bonds (as shown in Fig. 1a).^54,55,57^ Strategically, the p32 protein binding peptide (AKRGARSTA) is linearly linked to the pro-apoptotic peptide (KLAKLAKKLAKLAK) through the phosphorylated tripeptide (glycine–phosphorylated serine–glycine). The p32 protein (HAPB1, gC1qR or C1qbp) is a mitochondrial protein which highly expresses on the cell surfaces of activated endothelial cells and various tumor cells.^58,59^ Therefore, the p32 protein binding peptide can improve the tumor accumulation ability of nanoparticles. For comparison, the NaGdF_4_@CPP NPs were also prepared by same strategy. After ligand exchange, there are negligible changes on morphology, size and crystalline nature of NaGdF_4_ nanoparticles (as shown in Fig. 1b–g). The successful exchange of OA with phosphopeptides (bp-peptide and/or CPP) was demonstrated FTIR and EDS. After conjugation with phosphopeptides, the nitrogen and phosphorus peaks are clearly observed at 0.39 keV and 1.99 keV in the EDS spectra of NaGdF_4_@bp-peptide NPs and NaGdF_4_@CPP NPs (as shown in Fig. S1 in ESI†). The antisymmetric bending mode of PO_4_^3−^ (1083 cm^−1^) and synthetic spectrum band of stretching vibration and bending vibration of PO_4_^3−^ (1657 cm^−1^) are clearly observed in FTIR spectra of NaGdF_4_@bp-peptide NPs and NaGdF_4_@CPP NPs (as shown in Fig. S2†). The results indicate that the phosphopeptides are successfully immobilized on the NaGdF_4_ nanoparticle surface. The NaGdF_4_@bp-peptide NPs exhibit slight positive surface charges (0.63 mV) in H_2_O because the bp-peptide has relatively high isoelectric points (PI). The slightly positive charged surface may enhance the cellular uptake of NaGdF_4_@bp-peptide NPs. The hydrodynamic diameter (HD) and zeta potential of NaGdF_4_@bp-peptide NPs are 269.5 nm and −0.31 mV in culture medium, while the HD and zeta potential of NaGdF_4_@CPP NPs are 110.1 nm and −5.23 mV in culture medium. The phenomenon suggests that the interactions of NaGdF_4_@bp-peptide NPs with components of culture medium are stronger than those of NaGdF_4_@CPP NPs with components of culture medium. However, the longitudinal relaxivity (*r*_1_) value (5.8 mM^−1^ s^−1^) of NaGdF_4_@bp-peptide NPs is lower than that (7.9 mM^−1^ s^−1^) of NaGdF_4_@CPP NPs (as shown in Fig. 2). The relative low *r*_1_ value of NaGdF_4_@bp-peptide NPs may due to the branch structure and rigidity of bp-peptide which prolongs the exchange rate of the intra-spherical water molecules around the Gd^3+^.^60,61^

### Interactions of NaGdF_4_@bp-peptide NPs with cells

**Fig. 3:**
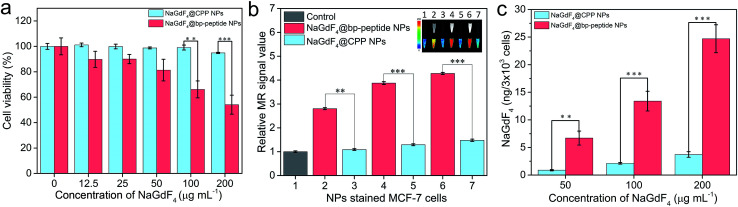
(a) Cell viabilities of MCF-7 cells after incubated with various concentrations of NaGdF_4_@bp-peptide NPs and NaGdF_4_@CPP NPs for 24 h, respectively. (b) The MR signal intensities and corresponding MR images (inset) of NaGdF_4_@bp-peptide NPs (2, 4 and 6) or NaGdF_4_@CPP NPs (3, 5 and 7) stained MCF-7 cells (the cells were incubated with 0 (1), 50 (2 and 3), 100 (4 and 5) and 200 (6 and 7) μg mL^−1^ NPs, respectively). (c) Amounts of Gd element in the nanoparticles stained MCF-7 cells. Error bars mean standard deviations (*n* = 5, **P < 0.01 or ***P < 0.001 from an analysis of variance with Tukey's post-test).

In order to test the cytotoxicity of as-prepared NaGdF_4_@bp-peptide NPs, the breast cancer cells (MCF-7) and human normal kidney tissue cells (293) were co-cultured the as-prepared NaGdF_4_@bp-peptide NPs. After incubated with up to 200 μg mL^−1^ NaGdF_4_@bp-peptide NPs for 24 h, the viability of MCF-7 cells is less than 60%, while viability of 293 cells is higher than 80% (as shown in Fig. 3a and S3†). The result suggests that NaGdF_4_@bp-peptide NPs have relatively low cytotoxicity to normal cells and could be used as antitumor agents. In addition, the NaGdF_4_@CPP NPs have low cytotoxicities to both of 293 cells and MCF-7 cells (as shown in Fig. 3a and S3†). The result indicates that the high cytotoxicity of NaGdF_4_@bp-peptide NPs towards MCF-7 cells originate from their ligands. The T_1_-weighted MR signal intensity of nanoparticle stained MCF-7 cell pellet is increased by increasing the concentration of nanoparticles in culture medium (as shown in Fig. 3b). Under same experimental condition, the MR signal intensity of NaGdF_4_@bp-peptide NPs stained MCF-7 cells is much stronger (≥2.9 times) that of NaGdF_4_@CPP NPs stained MCF-7 cells. In addition, the cellular internalization amount of NaGdF_4_@bp-peptide NPs is higher (≥6.7 times) than that of NaGdF_4_@CPP NPs (as shown in Fig. 3c). These results demonstrate that the binding affinity of NaGdF_4_@bp-peptide NPs with MCF-7 cells is much higher than that of NaGdF_4_@CPP NPs with MCF-7 cells.

### *In vivo* toxicity investigation

**Fig. 4:**
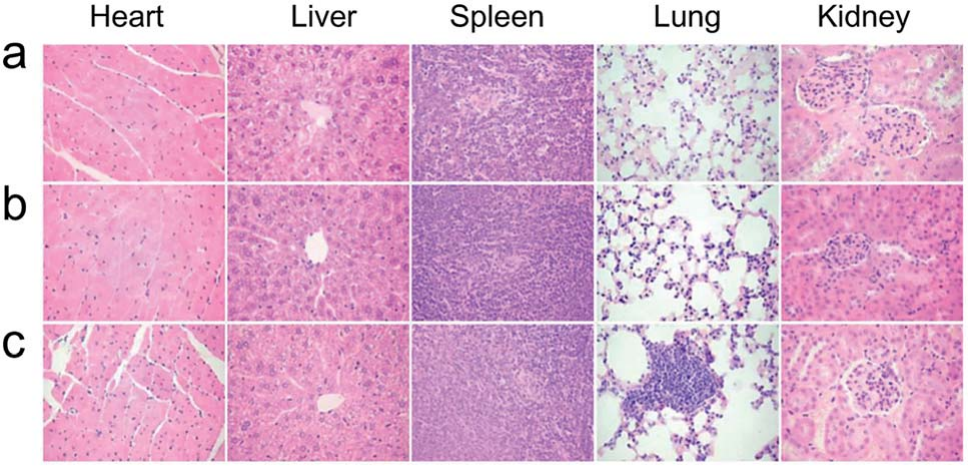
Histology analysis (H&E staining) of mice at 31 day post-injection. (a) Control, (b) NaGdF_4_@CPP NPs and (c) NaGdF_4_@bp-peptide NPs (10 mg Gd per kg body weight), respectively.

The healthy Kunming mice were intravenously injected a single dose (10 mg kg^−1^ of Gd) of NaGdF_4_@bp-peptide NPs or NaGdF_4_@CPP NPs, respectively. The blood routine analysis was used to test acute toxicities of nanoparticles. At 1 day post-injection, blood platelet count of NaGdF_4_@bp-peptide NPs treated mice is higher (2.44 times) than those of NaGdF_4_@CPP NPs treated mice and untreated mice, while other blood routine indicators of NaGdF_4_@bp-peptide NPs treated mice are comparable to those of NaGdF_4_@CPP NPs treated mice and untreated mice (as shown in Table S1†). The blood platelet count of NaGdF_4_@bp-peptide NPs treated mice is decreased with increasing the post-injection time. There is little difference in blood components among treated groups and control group at 7 day of post-injection. The long-term *in vivo* toxicities of nanoparticles were evaluated by monitoring the body weight changes of mice, histology analysis of major organs, and blood biochemical assays at 31 day of post-injection. As shown in Fig. S4,† the body weights of mice in all tested groups were increased steadily as the time prolonged. Comparing with the untreated mice, the main organs (*e.g.*, heart, liver, spleen, lung, kidneys) of NaGdF_4_@bp-peptide NPs or NaGdF_4_@CPP NPs treated mice exhibit little abnormalities or lesions (as shown in Fig. 4). For blood biochemical assays, there is negligible difference among untreated mice and NaGdF_4_@bp-peptide NPs (10 mg Gd per kg body weight) treated mice at 31 day of post-injection (as shown in Table S1†). The results further confirm the good biocompatibility of NaGdF_4_@bp-peptide NPs. The results of *in vivo* acute and chronic toxicity analysis suggest that the NaGdF_4_@bp-peptide NPs have reasonable biocompatibility.

### *In vivo* tumor-targeting capacity of NaGdF_4_@bp-peptide NPs

**Fig. 5:**
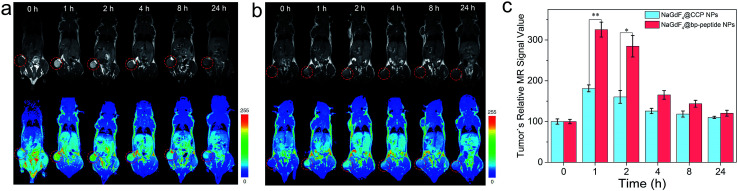
*In vivo* T_1_-weighted MR images and pseudo-color images of Balb/c mice bearing MCF-7 tumors after intravenous injection of (a) NaGdF_4_@bp-peptide NPs and (b) NaGdF_4_@CPP NPs (10 mg Gd per kg body weight) at different timed intervals (pre-injection (0), 1, 2, 4, 8 and 24 h post-injection), respectively. (c) Corresponding data analysis of MR measurements. The tumor sites were marked by circles. Error bars mean standard deviations (*n* = 3, *P < 0.05 or **P < 0.01 from an analysis of variance with Tukey's post-test).

The MCF-7 tumor-bearing Balb/c nude mice were used to investigate the tumor-targeting capacities of NaGdF_4_@bp-peptide NPs and NaGdF_4_@CPP NPs. The nanoparticles were intravenously injected into mice through the tail vein, respectively. The T_1_-weighted MR images of mouse were recorded at different time points (pre-injection (0), 1, 2, 4, 8 and 24 h) of post-injection. As expected, strongly positive MR contrast enhancement in the tumor sites are observed within 0 to 24 h post-injection of NaGdF_4_@bp-peptide NPs, and the maximum MR signal enhancement (3.25 times) was achieved at 1 h post injection (as shown in Fig. 5a and c). The NaGdF_4_@CPP NPs show relatively poor MR contrast enhancement in the tumor site (as shown in Fig. 5b and c). The result of *in vivo* MRI indicates that NaGdF_4_@bp-peptide NPs have good tumor-targeting capacity. The relative strong MCF-7 tumor-targeting capacity of NaGdF_4_@bp-peptide NPs may due to high binding affinity of bp-peptide with MCF-7 cells.

**Fig. 6:**
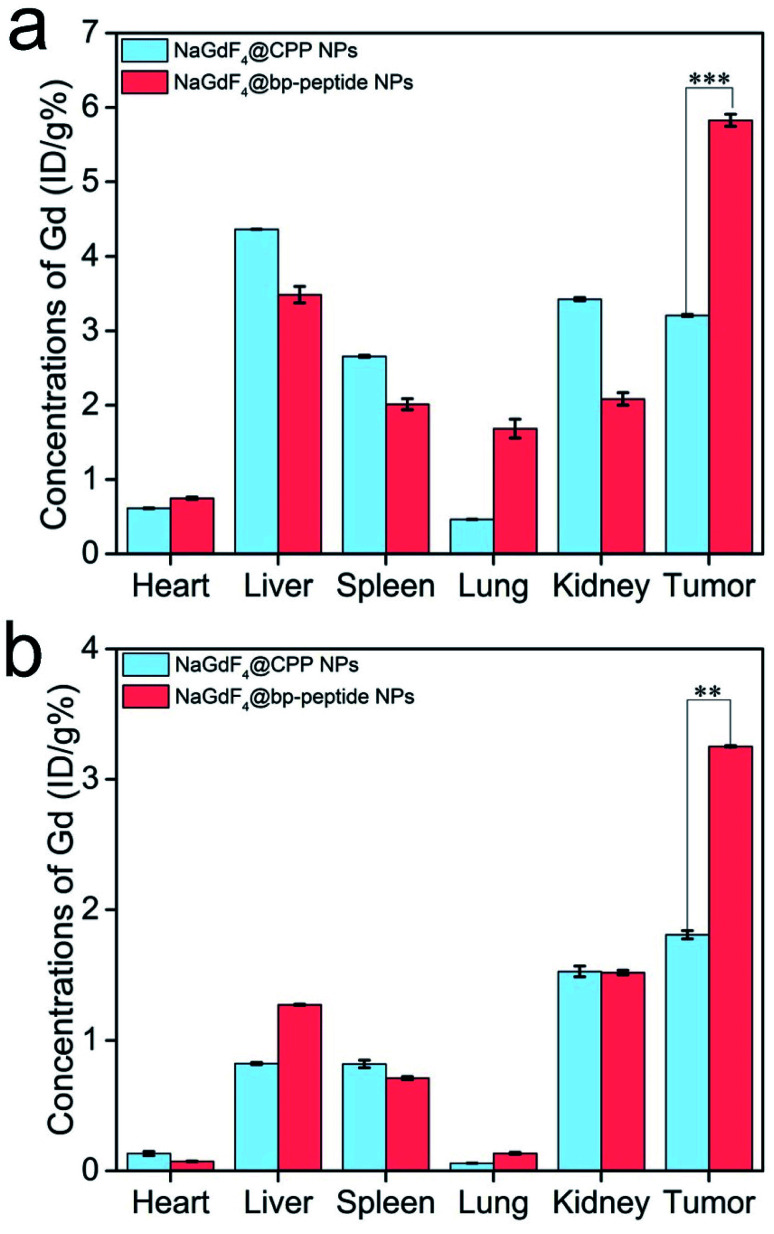
Total amounts of Gd element in the main organs and tumors of mice at 1 h (a) and 24 h (b) post-injection of NaGdF_4_@bp-peptide NPs or NaGdF_4_@CPP NPs, respectively. Error bars mean standard deviations (*n* = 3, **P < 0.01 or ***P < 0.001 from an analysis of variance with Tukey's post-test).

For further confirming tumor-targeting capacity of NaGdF_4_@bp-peptide NPs, the MCF-7 tumor-bearing Balb/c nude mice were sacrificed at 1 h or 24 h post-injection, respectively. The main organs and tumors of mice were collected, and the total amounts of Gd element in these tissues were measured by ICP-MS. As shown in Fig. 6, large amounts of Gd element were found in liver, spleen, kidneys and tumor. The Gd amounts in these organs at 1 h post-injection are higher than the Gd amounts in these organs at 24 h post-injection. The result indicates that both of NaGdF_4_@bp-peptide NPs and NaGdF_4_@CPP NPs are mainly accumulated in the liver, spleen, kidneys and tumor, and gradually excreted from body by hepatic and renal clearance pathways. Notably, the Gd amount in tumor of NaGdF_4_@bp-peptide NPs treated mouse is higher (1.82 times at 1 h post-injection and 1.78 times at 24 post-injection) than the Gd amount in tumor of NaGdF_4_@CPP NPs treated mouse. The result demonstrates that NaGdF_4_@bp-peptide NPs have excellent tumor-targeting capacity.

### *In vivo* antitumor efficacy of NaGdF_4_@bp-peptide NPs

**Fig. 7:**
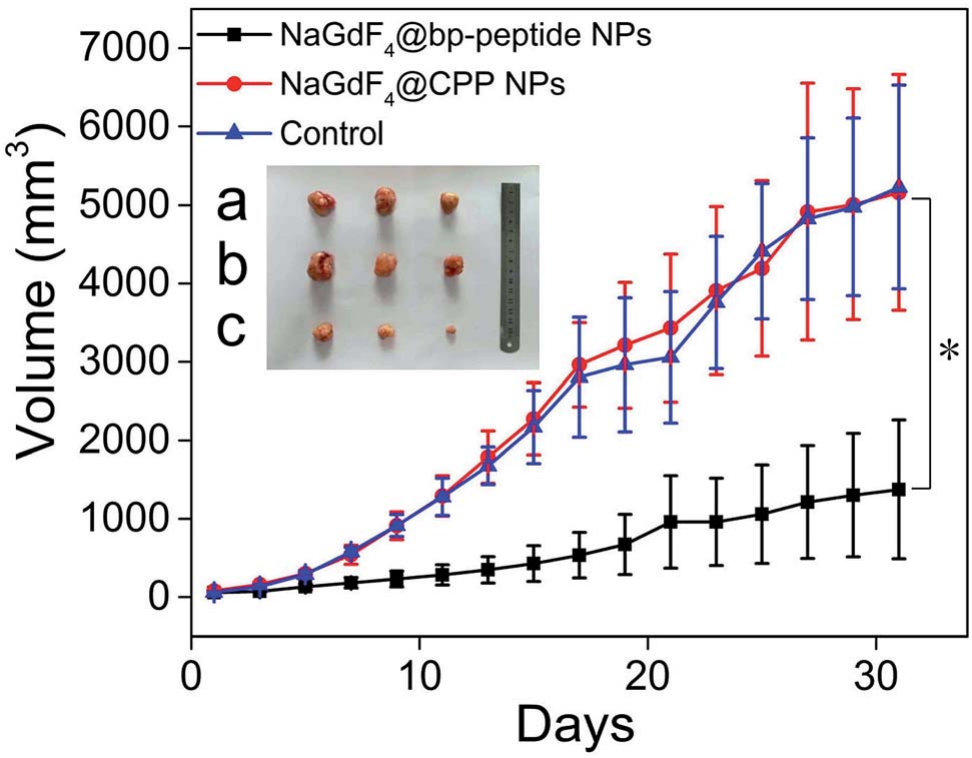
Tumor growth curves of Balb/c nude mice after different intravenous treatments. Inset digital photographs excised tumors, (a) control, (b) NaGdF_4_@CPP NPs and (c) NaGdF_4_@bp-peptide NPs (10 mg Gd per kg body weight), respectively. Error bar mean standard deviations (*n* = 3, *P < 0.05 from an analysis of variance with Tukey's post-test).

The MCF-7 tumor-bearing Balb/c nude mice were divided randomly into three groups (*n* = 3), control group, NaGdF_4_@bp-peptide NPs group, and NaGdF_4_@CPP NPs, which were treated by 100 μL PBS, and single dose (10 mg Gd per kg body weight) of NaGdF_4_@bp-peptide NPs and NaGdF_4_@CPP NPs through tail vein. After the different treatments were administered, the tumor sizes were measured with calipers every 2 days. Comparison with the PBS treated and NaGdF_4_@CPP NPs treated mice, the NaGdF_4_@bp-peptide NPs treated mice exhibit a delay in the tumor growth (as shown in Fig. 7 and S5†). The average tumor volume (1375 ± 885 mm^3^) of NaGdF_4_@bp-peptide NPs treated mice is much smaller than those of PBS treated (5229 ± 1296 mm^3^) and NaGdF_4_@CPP NPs treated (5160 ± 1501 mm^3^) mice. This result suggested that the NaGdF_4_@bp-peptide NPs have good antitumor bioactivity with the IRT as high as 73.7%.

## Conclusions

In summary, taking the advantage of strong interaction of phosphonate group with Gd^3+^, a new theranostic nanosystem (NaGdF_4_@bp-peptide NPs) has been constructed consisting of a NaGdF_4_ NPs core as the T_1_-weighted MR contrast agent and a peptide as the active-tumor targeting ligand as well as antitumor agent. *In vitro* and *in vivo* experiments demonstrate that the as-prepared NaGdF_4_@bp-peptide NPs have reasonable biocompatibility and excellent tumor targeting capacity. Furthermore, NaGdF_4_@bp-peptide NPs exhibit good anticancer efficacy with the IRT as high as 73.7% in subcutaneous MCF-7 breast tumor Balb/c nude mouse models. Owing to the diverse biological functionalities of peptides, this work provides us a facile preparation method to fabricate theranostic nanoparticles which have potential as anticancer agents for molecular imaging guided therapy.

## Conflicts of interest

There are no conflicts to declare.

## Supplementary Material

RA-009-C9RA02135C-s001
