# Supplementary material for: Peptide-functionalized NaGdF4 nanoparticles for tumor-targeted magnetic resonance imaging and effective therapy
Source: RSC Adv. 2019 May 31;9(30):17093–100. doi: 10.1039/c9ra02135c (PMC9064544; doi:10.1039/c9ra02135c)
Supplement: RA-009-C9RA02135C-s001 [file RA-009-C9RA02135C-s001.pdf]

Electronic Supplementary Material (ESI) for RSC Advances.
This journal is © The Royal Society of Chemistry 2019

Electronic Supplementary Information (ESI) for:

Peptide-Functionalized $NaGdF_4$ Nanoparticles for Tumor-Targeted Magnetic Resonance Imaging and Effective Therapy

Yixin Chen[a], Yu Fu[a], Xiaodong Li[a], Hongda Chen*[b], Zhenxin Wang,[b] Huimao Zhang*[a]

[a]Department of Radiology, the First Hospital of Jilin University, Changchun 130021, P. R. China. E-mail: huimaozhanglinda@163.com (HZ); Fax: (+86) 431-88783300.

[b]State Key Laboratory of Electroanalytical Chemistry, Changchun Institute of Applied Chemistry, Chinese Academy of Sciences, Changchun, 130022, P. R. China. E-mail: chenhongda@ciac.ac.cn (HC); Fax: (+86) 431-85262756.

Contents

1. **Additional Figures S1-S5.**

2. **Additional Table S1.**

1. Additional Figures

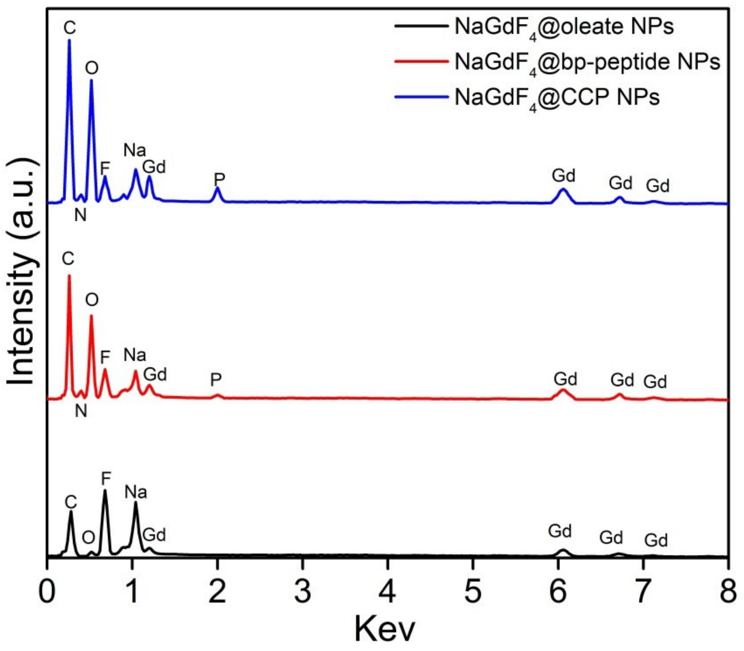

Fig. S1 EDS spectra of as-prepared nanoparticles.

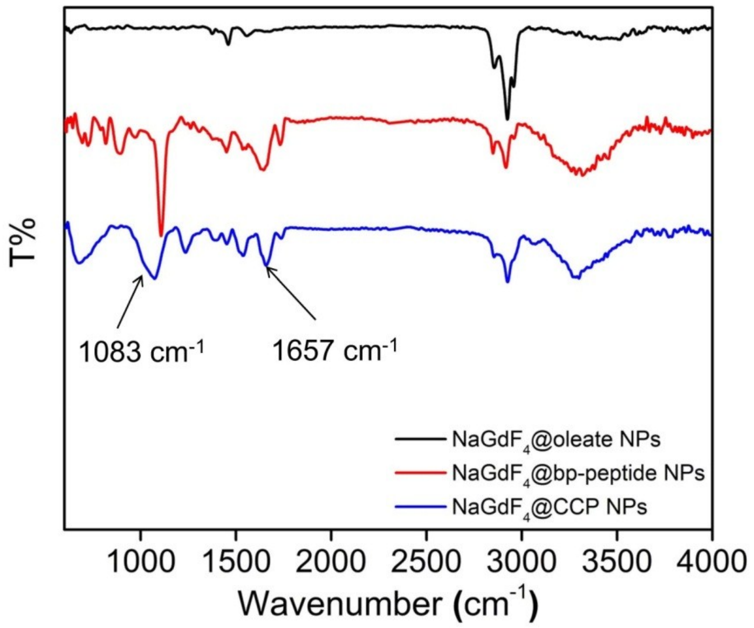

Fig. S2 FTIR spectra of as-prepared nanoparticles.

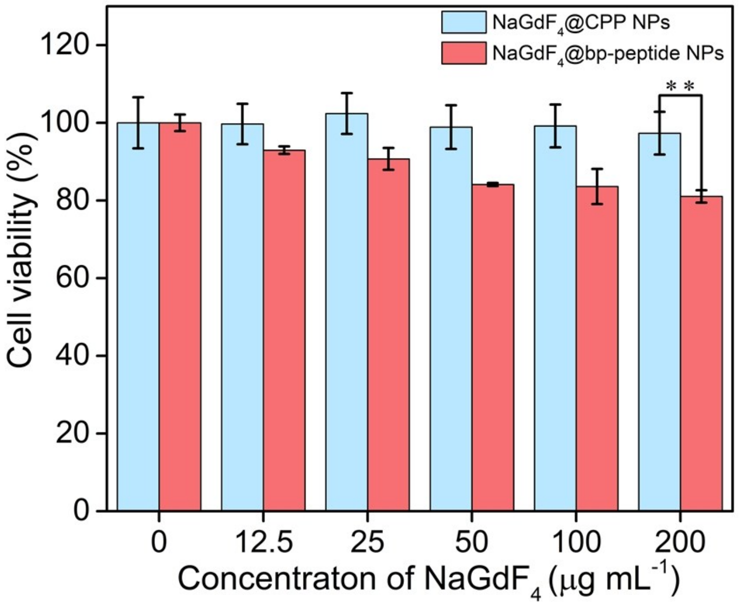

Fig. S3 Cell viabilities of 293 cells after incubated with various concentrations of $NaGdF_4$@bp-peptide NPs and $NaGdF_4$@CPP NPs for 24 h, respectively (n=5, **$P<0.01$ from an analysis of variance with Tukey's post-test).

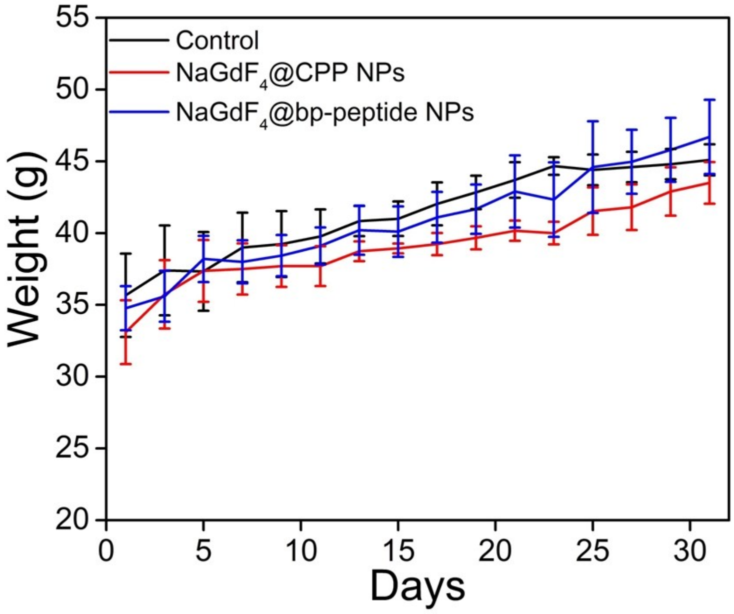

Fig. S4 Body weight changes of the health Kunming mice with and without intravenous injection of $NaGdF_4$@bp-peptide NPs and $NaGdF_4$@CPP NPs (10 mg Gd kg^{-1} body weight) versus time.

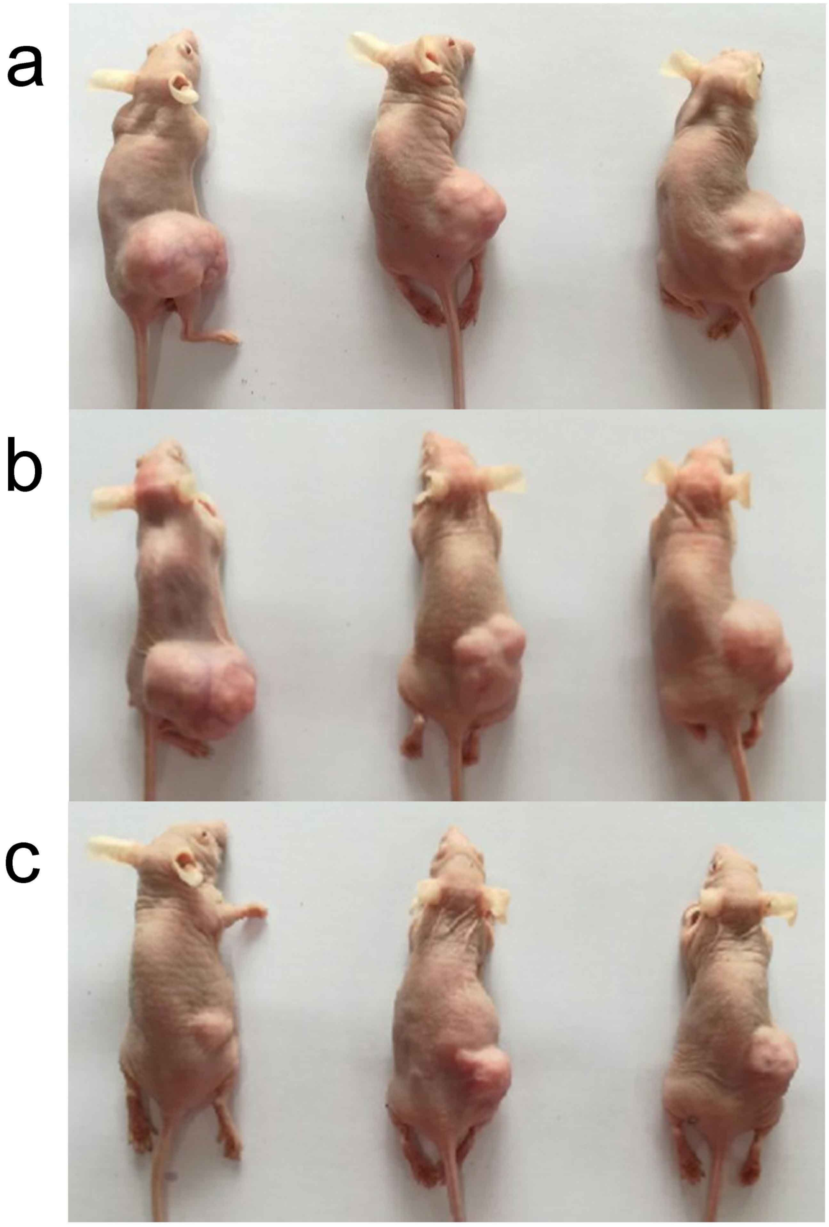

Fig. S5 Digital photographs of Balb/c nude mice after different intravenous treatments. (a) Control, (b) $NaGdF_4$@CPP NPs and (c) $NaGdF_4$@bp-peptide NPs (10 mg Gd kg^{-1} body weight), respectively.

2. Additional Table

Table S1. Blood routine analysis of mice at 1 day, 7 day and 31 day of post-injection.

**Test (1 day)**	**Units**	**Control**	**$NaGdF_4$@CPP NPs**	**$NaGdF_4$@bp-peptide NPs**
**WBC**	$\times 10^9$/L	4.50	5.70	3.20
**RBC**	$\times 10^{12}$/L	7.75	8.14	6.44
**HGB**	g/L	108	118	93
**LY**	%	62.3	65.6	69.9
**MCH**	pg	13.9	14.4	14.4
**MCHC**	g/L	350	332	349
**MCV**	fL	39.8	43.7	41.4
**PLT**	$\times 10^9$/L	446	442	1.08×10^3
**PDW**	fL	14.6	14.9	14.0

**Test(7 day)**	**Units**	**Control**	**$NaGdF_4$@CPP NPs**	**$NaGdF_4$@bp-peptide NPs**
**WBC**	$\times 10^9$/L	6.30	6.40	5.90
**RBC**	$\times 10^{12}$/L	8.70	8.54	8.83
**HGB**	g/L	135	120	130
**LY**	%	60.3	61.8	62.9
**MCH**	pg	15.5	14.0	14.7
**MCHC**	g/L	338	353	344
**MCV**	fL	45.9	39.7	42.8
**PLT**	$\times 10^9$/L	528	490	563
**PDW**	fL	14.0	15.0	13.2

**Test (31 day)**	**Units**	**Control**	**$NaGdF_4$@CPP NPs**	**$NaGdF_4$@bp-peptide NPs**
**WBC**	$\times 10^9$/L	4.90	4.80	3.90
**RBC**	$\times 10^{12}$/L	6.94	7.24	6.15
**HGB**	g/L	112	110	98.0
**LY**	%	60.5	62.3	62.8
**MCH**	pg	11.8	12.5	13.7
**MCHC**	g/L	342	314	338
**MCV**	fL	35.8	42.8	40.8
**PLT**	$\times 10^9$/L	441	435	477
**PDW**	fL	14.9	13.8	13.6
